# Anesthetics and plants: no pain, no brain, and therefore no consciousness

**DOI:** 10.1007/s00709-020-01550-9

**Published:** 2020-09-02

**Authors:** Andreas Draguhn, Jon M. Mallatt, David G. Robinson

**Affiliations:** 1 Institute for Physiology and Pathophysiology, Medical Faculty, University of Heidelberg, 69120 Heidelberg, Germany; 2 The University of Washington WWAMI Medical Education Program, The University of Idaho, Moscow, ID 83844 USA; 3 Centre for Organismal Studies, University of Heidelberg, Im Neuenheimer Feld 230, D-69120 Heidelberg, Germany

**Keywords:** General anesthetics, Sleep, Ion channels, Perception, Cognition

## Abstract

Plants have a rich variety of interactions with their environment, including adaptive responses mediated by electrical signaling. This has prompted claims that information processing in plants is similar to that in animals and, hence, that plants are conscious, intelligent organisms. In several recent reports, the facts that general anesthetics cause plants to lose their sensory responses and behaviors have been taken as support for such beliefs. These lipophilic substances, however, alter multiple molecular, cellular, and systemic functions in almost every organism. In humans and other animals with complex brains, they eliminate the experience of pain and disrupt consciousness. The question therefore arises: do plants feel pain and have consciousness? In this review, we discuss what can be learned from the effects of anesthetics in plants. For this, we describe the mechanisms and structural prerequisites for pain sensations in animals and show that plants lack the neural anatomy and all behaviors that would indicate pain. By explaining the ubiquitous and diverse effects of anesthetics, we discuss whether these substances provide any empirical or logical evidence for “plant consciousness” and whether it makes sense to study the effects of anesthetics on plants for this purpose. In both cases, the answer is a resounding no.

## Introduction

With the aim of eliminating pain, memory, movements, and conscious experience during operations, volatile anesthetics were introduced into medical practice more than 170 years ago. Interestingly plants also react to these substances as originally demonstrated by Claude Bernard in 1878, causing him to claim that “… plants and animals must share common biological essence that must be disrupted by anesthetics” (Bernard 1878; Grémiaux et al. 2014). In the following 100 years, reversible inhibitory effects of anesthetics on various aspects of plant growth and motility were recorded (e.g., Bancroft and Rutzler 1931; Bünning 1934; Taylorson 1982), with little indication as to their mode of action. With the introduction of the plant neurobiology concept (Brenner et al. 2006), the notion that plants are conscious organisms has become more popular, especially in the popular press (e.g., Trewavas and Baluska 2011; Calvo et al. 2017; Gagliano et al. 2017; Trewavas 2017; Mancuso 2018). Consciousness is defined as the capacity of an organism to have experiences, to feel sensations, and to carry out voluntary behaviors (Nagel 1974; Mallatt et al. 2020). Because anesthetics induce an unconscious state in humans, their effects on plants have been taken to indicate the existence of consciousness in plants, including the conscious perception of pain (Baluška 2016). This apparent similarity has prompted plant neurobiologists to analyze anesthetic’s effects more closely (Yokawa et al. 2018, 2019; Pavlovič et al. 2020). Among the reversible effects they recorded are the loss of responsiveness to external stimuli, cessation of phototactic and plant organ movements, inhibition of seed germination and of accumulation of chlorophyll, alteration of ROS homeostasis, impairment of jasmonate signaling, blockage of action potentials, and inhibition of endocytic vesicle recycling. The impairment of jasmonate signaling has received special attention recently (Pavlovič et al. 2020) because jasmonate is involved in long-distance electrical communication in plants. Therefore, its disruption has been taken as evidence for systemic effects of anesthetics similar to the disruption of coordinated information processing in the mammalian brain (Trewavas et al. 2020). Together, the effects of anesthetics on local and systemic functions of plants have evolved into a major argument for similarities between plants and animals with respect to consciousness. Due to these proclaimed similarities, plants are being advertised as model organisms for studying anesthetics (Yokawa et al. 2018, 2019; Baluška and Reber 2019).

Volatile general anesthetics include diethyl ether, isoflurane, sevoflurane, halothane, and more. Each affects a large variety of molecular targets, many of them being present—in homologous or analogous forms—in all living phyla (Kelz and Mashour 2019). The best studied of these general effects, demonstrated in organisms ranging from bacteria to plants to animals, is altering the functions of many kinds of protein receptors and ion channels in cell membranes (Hemmings et al. 2019). In animals, anesthetics especially affect *neurons*, leading to specific effects in their nervous systems (which plants lack). As an overall neural effect, anesthetics disrupt coordinated activity patterns within and between neuronal networks, suppressing sensation, action, and conscious experience (Kelz and Mashour 2019; Akeju and Brown 2017). The underlying mechanisms are not fully understood but the effects on ion channels seem to be a large part, and all known molecular and cellular effects converge on a disruption of synaptic transmission and electrical activity in neuronal networks. These effects are most likely mediated by the potentiation of inhibitory and the suppression of excitatory postsynaptic transmitter receptors, a general reduction of neurotransmitter release, and reduced intrinsic neuronal excitability. Further actions on the cytoskeleton and mitochondrial complex I are believed to add to the impairment of synaptic transmission (Kelz and Mashour 2019). As a result, network- and system-level activity of the brain is altered to end consciousness, in a way that shows some similarities, but also profound differences to sleep (Akeju and Brown 2017). Depending on their chemical structures and concentrations, different anesthetics affect different neural subsystems, leading to specific effects and prompting differential clinical uses.

We thus have to distinguish the general effects of anesthetics, which likely apply to all living matter, from the specific effects on neuronal mechanisms in the brain of humans and animals. The latter explain the specific and reversible disruption of perception and consciousness, which occur in animals that possess the required brain structures. One of the most distinct actions of anesthetics in mammals is, of course, mitigating or abolishing the sensation of pain (Rowley et al. 2017). This is not only a primary reason for their clinical use, it is also closely linked to consciousness (Feinberg and Mallatt 2016; Walters and Williams 2019). Therefore, pain provides a handle to study consciousness in nonverbal organisms.

Those who advocate the effects of anesthetics on plants as proof for their consciousness (Baluška et al. 2016; Yokawa and Baluška 2018; Yokawa et al. 2018; Pavlovič et al. 2020; Trewavas et al. 2020) challenge prevailing concepts of the *neuronal* basis of this complex state. Pain-relieving effects of such substances in animals and humans have been used to infer subjective pain experiences in plants (p. 6 in Baluška et al. 2016). This argument raises severe semantic, conceptual, and scientific problems. We will therefore summarize current knowledge on pain in humans and animals and critically ask whether the effects of anesthetics in plants suggest they also feel pain. We will then extend our arguments to the question of consciousness and ask whether anesthetics provide any evidence for its existence in plants. As a whole, we hope to show the irrelevance of studying anesthetics in plants for promoting concepts such as plant sentience, plant cognition, or plant consciousness.

## Pain in humans: a complex experience and its neural basis

As defined by the International Association for the Study of Pain (https://www.iasp-pain.org/terminology?navItemNumber=576#Pain), pain is “an unpleasant sensory and emotional experience associated with actual or potential tissue damage.” Thus, as originally described by the eminent British neurophysiologist and Nobel Prize winner, Charles Scott Sherrington, pain is a subjective experience following on from a physiological process involving neuronal stimulation by cues indicating tissue damage. This peripheral physiological process, termed “nociception” (from the Latin verb *nocere*, which means to harm), is not identical to pain. It only triggers electrical activity in nociceptive neural pathways and widespread networks of the brain that then give rise to the perception of a lesion, adequate reactions, and the subjective experience of pain. Pain is a particularly salient and emotional experience that almost inevitably demands our full attention. This is why it has a central role in the study of consciousness (Feinberg and Mallatt 2016; Harnad 2016; Walters and Williams 2019).

In mammals, the detection of harmful chemical, mechanical, or thermal cues (collectively termed noxious stimuli) is accomplished by nerve endings (nociceptors) found in the skin, joints, and multiple other organs. Nociceptors express a large variety of membrane-bound receptor molecules for the different types of potentially damaging stimuli (e.g., cuts, extremes of pH, and temperature). The nociceptors come in diverse forms—some triggering the sharp, well-localized sensation of pain immediately following a pinprick; others the delayed, nasty sensation of a burning pain seconds after we hurt ourselves; and others the sensation of itch. They also carry receptors for inflammation molecules that enhance their reactivity, as we all know from the hypersensitivity at or around an infected fingernail or any other local irritation. With this molecular toolkit, nociceptors “translate” damaging stimuli into electrical signals. These signals, called action potentials, are fast fluctuations of the membrane potential travelling along the nerve fiber of the nociceptor neuron, reaching the spinal cord and then transferring the excitation to secondary neurons in the central nervous system. From there, activity is distributed within a large, widespread network of different brain regions. It is the combined activation pattern of these brain regions, called “pain network” or “pain matrix,” that does finally give rise to the complex, unpleasant sensation of pain.

Looking into the locations and functions of the different brain areas for pain processing is instructive to understand further physiological and psychological features of pain (Craig 2003; Almeida et al. 2004). Nerve fibers from the spinal cord send branches to many different parts of the brain stem and forebrain. One central hub in the lower forebrain, called the thalamus, distributes the activity to several distinct regions of the cerebral cortex that convey different aspects or “components” of the pain sensation. We can attribute each component to one major brain region, notwithstanding that this is an oversimplification that ignores the distributed, network-like character of all processes in the brain. Actually, there is some debate over whether the sensation of pain entirely emerges up in the cerebral cortex, or whether it instead emerges in raw form below the cortex (perhaps partly in the thalamus) with the cerebral cortex only adding certain components and modulating the pain, e.g., when we cognitively magnify or repress it (Devor 2016; Key 2016). In any case, as a first approximation, we can distinguish and localize the following components:

- The discriminative component: Where is the lesion on the body, and what were the nature and duration of the injury? This can be mostly attributed to the somatosensory area of the cerebral cortex, a region where also other body sensations (touch, pressure, temperature, etc.) are processed.
- The cognitive component: What does the pain mean for me, how dangerous is it, and how should I react? This aspect is important for our behavioral response, and it depends heavily on the function of the prefrontal cortex, the most anterior part of the cerebral cortex.
- The affective component: This is a scientific name for what we all know from experience—pain hurts and causes suffering. It is the aspect of pain that mostly concerns us here and the subject of the above-mentioned debate over whether pain arises in the cortex or subcortically. The dominant view is that pain affects arise in the parts of the cerebral cortex called the cingulate gyrus, which is a round, belt-shaped ridge on the medial side of the cerebral hemisphere, and the insula, which is a recessed island of the lateral cortex deep to your ear. The alternate view, based on the clinical findings that damage to these two cortical regions does not eliminate the pain but just changes its intensity or the attention one pays to it, is that a significant affective pain component arises subcortically, perhaps partly in the amygdala (see below).
- Stress and fear: These reactions are mediated by an almond-shaped nucleus in the depth of the temporal lobe called the “amygdala.” This area has gained much interest in recent years as it is involved in processing all sorts of fear, including pathological forms of anxiety, e.g., claustrophobia (Vadakkan and Siddiqui 2019).
- Finally, pain causes multiple nonconscious, physiological reactions of the body, beginning with fast withdrawal reflexes (organized within the spinal cord), general arousal, and activation of the sympathetic nervous system for “fight or flight” (mediated by the nuclei within the brain stem), and stress-related hormonal changes (e.g., an increase in cortisol, mediated by a forebrain region underneath the thalamus, called hypothalamus).

The large variety, wide distribution, and functional specialization of different brain areas involved in the generation of pain underline the richness and complexity of this conscious state. This is also visible from the diverse, often challenging clinical situations of severe or chronic pain. Pain (real pain!) can occur by malfunction of the pain system itself, in the complete absence of any lesion. Pain components can dissociate, meaning one can have pain but it does not hurt, or one can suffer from pain but cannot localize or describe it. Interestingly, pain is subject to all of the neuronal mechanisms involved in learning processes: synaptic plasticity, extension of pain-related brain regions, and strengthening of pain sensations by rewards. As a result, pain can become chronic and turn into a genuine pain disease where the pain processing system has become a problem, rather than a protective mechanism. Finally, organ recovery from brain-dead patients may and can be done without anesthesia. This legal and ethical consideration reflects the firm causal link between brain function and pain experience. No brain, no pain.

Why have we described human pain so thoroughly in an article about plants? It was to show that pain processing, if it is anything like our human experience, is a complex phenomenon involving neurons and specialized brain regions—and plants have neither.

## Pain in nonhuman animals

All mammals have all the components of the human pain system, from the nociceptors, to the pain pathways, subcortical brain regions, and the various pain-related areas of the cerebral cortex. Thus, it is widely accepted that all mammals can experience pain. None of the other vertebrates—birds, reptiles, amphibians, or fish—has a mammal-like cortex in their cerebrum. This has prompted researchers who assert that pain requires a cerebral cortex to state that non-mammalian vertebrates do not feel pain (Key 2016). This statement is controversial, however, because all these vertebrates have the subcortical brain structures involved with nociceptive processing; thus, researchers who assert that pain arises subcortically say all vertebrates can experience pain (Feinberg and Mallatt 2016, Chapter 8). The argument that pain needs a cerebral cortex has also received much criticism because traits can evolve from multiple alternate structures to the same end (Sneddon and Leach 2016). In birds, for example, the enlarged cerebral hemispheres have analogous regions to the mammalian neocortex, in line with their highly advanced cognitive abilities (Güntürkün and Bugnyar 2016). Thus, most researchers and government policies that regulate humane treatment of laboratory animals say that all vertebrates feel pain (Committee for Recognition and Alleviation of Pain 2009; Mikhalevich and Powell 2020).

What about invertebrates? Some of them have complex brains and behaviors, namely, the arthropods such as insects, lobsters, and spiders and cephalopods such as octopuses and squid. These invertebrates have nociceptors, but their brains evolved independently of vertebrate brains and are quite different. Furthermore, the nociceptive processing parts of their brains have not yet been located. To judge if they are conscious, it is necessary to look for the *behaviors* that associate with pain in the vertebrates and see if these invertebrates perform them. The pain-indicating behaviors are operant learning, from experience, of strategies to avoid noxious stimuli; learning to avoid a place where a noxious stimulus was formerly presented (conditioned place aversion); specific changes in behavior such as rubbing and guarding the wound; and self-delivery of analgesic pain relievers (Feinberg and Mallatt 2016, pp.150-153; Walters 2018; Sneddon 2019). Among invertebrate animals, only the cephalopods and many arthropods pass these criteria, and increasing numbers of investigators accept that they feel pain (Elwood 2020; Mikhalevich and Powell 2020).

Again, what does this have to do with plant pain? No operant learning or conditioned place aversion has ever been demonstrated in plants so they seem to fail those pain tests.

## Pain in plants?

Now we can examine more systematically whether plants feel pain. For this, there are two basic questions:

- Do plants have nociceptive cells and molecular receptors for noxious stimuli such as ASICs (acid sensing ion channels) or TRPs (transient receptor potential channels), the two most frequently occurring nociceptors in animals (Smith and Lewin 2009)? In regard to nociceptive sensory cells, the answer is definitely no. In regard to the receptor molecules, the answer is most probably not, but one should bear in mind that plants have receptors and ion channels with similarities to the molecular constituents of animal nociceptive systems. Among these are plant ion channels that alter their gating with pH, similar to ion channels in animals within and outside the nociceptive system. For example, both of the guard cell K^+^ channel families (gated outwardly rectifying potassium channel, GORK; gated inwardly rectifying potassium channel, KAT) are sensitive to pH (Dietrich et al. 2001), as are many mammalian K+ channels (Sepúlveda et al. 2015). Likewise, both plants (Hamant and Haswell 2017) and animals (Jin et al. 2020) have mechanoreceptors. In animals, these receptors serve multiple functions from mediating touch to hearing, posture, and balance. While some mechanoreceptors *in animals* monitor mechanical damage and are thus nociceptive, this does not justify any claim for a nociceptive sensory system in plants just by analogy.

b) Do plants have a system for integration and experience of damaging stimuli, similar to the complex, highly specialized pain processing network in animals? Definitely not: we reiterate that plants lack both neurons and a brain or any other substrate for central representations of inner states. They therefore cannot experience pain. Advocates of consciousness and cognition in plants point out, however, that plants react to damaging cues with widespread electrical and chemical signals, resembling a coordinated reaction (van Bel et al. 2014; Gallé et al. 2015). Plants do indeed respond to burning injuries and destructive wounding by “slow wave membrane potentials” (Nguyen et al. 2018; Lew et al. 2020), by accumulating jasmonate (Pavlovič et al. 2020) and releasing various volatile substances (Baluška et al. 2016). None of these processes has, however, any similarity to the initiation and distributed processing of pain in animals. An important limitation of electrical signaling in plants is that, as far as we know, it is all one way without any feedback messaging to allow signal exchanges (R. Hedrich, personal communication). Thus, plants have no coordinated network nor center for integrating the specific cues and reactions to damage, in sharp contrast to pain-experiencing animals and humans.

Among the plant neurobiologists, Baluška (2016) gave the fullest consideration of why plants might experience pain. He provided five possible reasons: (1) stressed plants are known to produce anesthetics, the major ones being ethylene and divinyl ether, and this could be to relieve the plant’s own pain; (2) plants express glutamate and GABA receptors, similar to animal’s neurons; (3) plant roots grow away from danger as if showing a plant version of negative feelings; (4) plants are sensitive to the behavior-suppressing effects of numerous *exogenous* anesthetics; (5) all living organisms may need pain states to survive in a dangerous world.

None of these reasons seems to hold up. The first argument that a plant makes anesthetics to relieve its own pain may indeed deserve further consideration and experimental investigation. Speaking against it, however, is that the ethylene produced by stress acts more like a plant *hormone* than an anesthetic, as it has only been shown to signal ordinary, physiological responses (tolerance to wounding, heat, cold, drought, salt); furthermore, ethylene is not only produced under stressful conditions that would require pain relief but also throughout the life cycle to regulate the plant’s growth, development, and senescence (Müller and Munné-Bosch 2015; Yang et al. 2015; Iqbal et al. 2017). Likewise, the other purported “anesthetic,” divinyl ether is tied to pathogen resistance, not to plant neurobiology (Stumpe et al. 2008; Fammartino et al. 2010). The second argument, that plants possess typical neurotransmitter receptors, is flawed as long as no evidence is produced for information processing in synaptically connected, neural-like networks in plants (more on this later). The third argument, that roots grow away from danger, refers to a merely physiological adaptation, namely, the avoidance response that is present in all organisms including prokaryotes (which most scientists consider to lack feelings: Mallatt and Feinberg 2017). Fourth, plants’ susceptibility to exogenous anesthetics is only exemplifying the universal, disengaging effects of these substances on all living organisms, which should not be confounded with their specific actions on nervous systems that affect pain perception, actions, consciousness, and memory (Kelz and Mashour 2019). The fifth argument, on the universality of pain states for all living matter, is nothing more than speculation, rooted in the nineteenth century vitalism (Bernard 1878; Perouansky 2012; Grémiaux et al. 2014).

## Anesthetics, consciousness, and sleep

General anesthetics not only block the sensation of pain in humans but also alter consciousness. This effect distinguishes them from local anesthetics, which inhibit the excitation of sensory nerve endings by binding to membrane-sodium channels, and from purely analgesic drugs, which act on different levels of the nociceptive system without affecting consciousness. Because the effects of anesthetics on plants are increasingly being used as arguments for plant consciousness, it is important to illuminate the respective mechanisms, which are, not surprisingly, tightly linked to complex functions within the central nervous system. The nonconscious state induced by most general anesthetics has some similarities to sleep, another state of altered consciousness. In popular descriptions, anesthesia is often described as putting the patient to sleep. In this section, we compare these two states for any insights into whether plants have consciousness.

Anesthetic substances can affect consciousness in different ways and degrees, varying from complete loss of consciousness to disconnected states of consciousness where external cues are excluded (resembling a dream experience) or even connected consciousness where some awareness of outer cues is preserved (Bonhomme et al. 2019). The underlying mechanism is not a global inhibition of all neuronal signaling but rather a loss of coordination (coupling) between neurons in specific brain areas (Akeju and Brown 2017; Kelz and Mashour 2019; Hudson 2020).

Here are some similarities and differences between the anesthetized and the sleeping brain. Both states involve specific and complex activity patterns in defined brain regions and are, thus, tightly linked to the electrical activity of the nervous tissue. Many anesthetics affect activity in sleep-regulating networks including the hypothalamus, arousal systems in the brain stem, and recurrent cortical-subcortical loops that are essential for consciousness (Bonhomme et al. 2019). In all cases, however, the resulting activity patterns under anesthesia deviate from the typical, brain-wide synchronous oscillations characteristic of non-rapid eye movement (non-REM) sleep as well as from the waking-like patterns that characterize REM sleep (Bonhomme et al. 2019; Akeju and Brown 2017).

Do plants sleep, and could anesthetizing them tell anything about this? Several advocates of plant consciousness have claimed plants do sleep (Bose 1927; Barlow 2015; Shepherd 2017; Lamme 2018). This claim can be clearly negated, however. In animals with a complex brain, sleep proceeds through highly specific activity patterns (Purves et al. 2017), including different brain states and phases that are accompanied by multiple cognitive (e.g., dreaming in mammals) and physiological (e.g., decreased muscle tone) reactions. Thus, sleep in the sense of altered consciousness requires a highly differentiated central nervous system. What we share with simpler animals like the worm *Caenorhabditis elegans* is a circadian alteration of nervous activity (Anafi et al. 2019). The quiescent phase can be—and often is—called “sleep”, but then it does not have any connotation of consciousness (i.e., of a nonconsciousness that wakes into consciousness). Similarly, plants follow circadian changes of activity. This is obvious in the case of photosynthetic light harvesting, which ceases during the night, and its rhythms involve metabolic, transcriptional, cellular, and system-level mechanisms. As Lefoulon et al. (2020) have recently shown for plants that have CAM (Crassulacean acid metabolism), the nocturnal cessation of anion channel activity in guard cells is due to a stoppage of channel protein synthesis—a physical rather than a mental (sleeping) explanation. In no way does the ubiquitous presence of the day-night cycle in organisms imply the presence of consciousness. There is no electrophysiological evidence that plants have a sleeping state similar to ours. Because plants do not sleep, they do not have the known-to-be-conscious experiences of REM sleep and waking up. And, without sleep, their responses to anesthetics cannot inform nervous aspects of sleep research.

## Anesthetics and plants

**Fig. 1:**
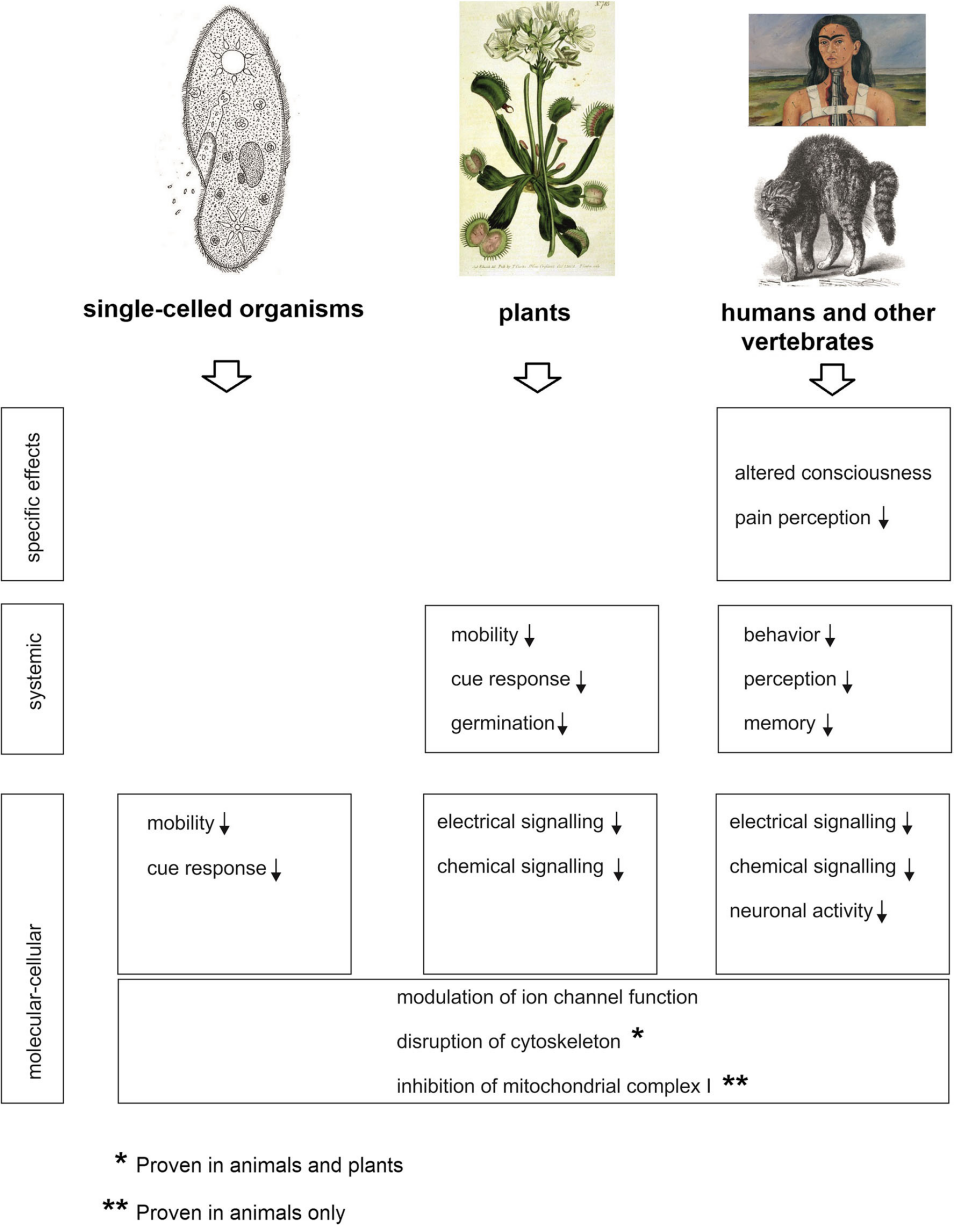
Effects of anesthetics in different taxa of organisms. Conserved effects are in the bottom rows (molecular-cellular) and grade to more taxon-specific effects in the middle and top rows. Note that the effects on mitochondrial complex I have only been shown for animals/humans but may well be present in plants and single-celled organisms (see main text). Sources of the illustrations are (1) http://www.biology-resources.com/drawing-paramecium.html (D.G. Mackean); (2) https:www.biologie-seite.de/Biologie/Venusfliegenfalle (William Curtis 1790); (3) Frieda Kahlo painting, 1944 (the broken column showing the results of her spinal surgery for a painful back injury); (4) a fearful cat. Charles Darwin (1872) The expression of emotion in man and animals. John Murray, London

Kelz and Mashour (2019) covered the many molecular targets of general anesthetics, emphasizing those targets that are conserved from single-celled organisms to humans (Fig. 1). As we mentioned, the best documented of these targets are many kinds of ion channels, of both the voltage-gated and ligand-gated types. These include “pLGIC” protein channels (pentameric ligand-gated ion channels) such as GABA_A_ (gamma-aminobutyric acid) receptors (Hemmings et al. 2019), Na+ channels (Barber et al. 2014), K+ channels (MacKinnon et al. 1998; Li et al. 2018), and Ca^2+^ channels. Additionally, the anesthetics probably target the lipid bilayers of cellular plasma membranes (Pavel et al. 2020). Many of anesthetics’ “neuron-specific” effects on animals are conserved effects that have been elaborated to disrupt electrical signaling in the nervous system. In their list of conserved effects, Kelz and Mashour (2019) also included disruption of microtubules of the cell skeleton and inhibition of mitochondrial complex I, although the authors only discussed these effects in animals.

Turning to the effects of anesthetics on *plants*, the inhibition of electrical signaling has been documented (Yokawa et al. 2018; Pavlovič et al. 2020) as has a disruption of microtubules (Dustin 2012). In addition, considering the basic similarity in structure of the mitochondrial complex I in mammals, yeast, and plants (Davies et al. 2018), it is highly likely that substances targeting this complex in mammals also do so in plants. Ion channels, microtubules, and correctly functioning mitochondria are so fundamental to the physiology of plants that their inhibition through anesthetic treatment will inevitably lead to a shutdown of cell function. Therefore, it seems that conserved, general effects of anesthetics could account entirely for plants’ responses to them, and no neuron-like effects need be proposed.

As mentioned, plants have glutamate and GABA signaling and receptors. Animal versions of these receptors are targets of anesthetics in the animal nervous systems, especially at neuron-to-neuron synapses where glutamate and GABA act as neurotransmitters (Kelz and Mashour 2019). From this, plant neurobiologists have inferred that plants’ susceptibility to anesthetics reveals that plants have the same types of “neuronal” processes involved in animal consciousness (Baluška 2016; Yokawa et al. 2018; Trewavas et al. 2020). As pointed out by Taiz et al. (2020), however, this is a giant leap in logic especially because plants do not have true neurons or any equivalents of synapses (claims for “plant synapses” being especially dubious: Robinson et al. 2018). A neurotransmitter-like function is questionable for *glutamate* in plants, where glutamate acts as a multipurpose and far-ranging signal for many different physiological processes, a high fraction of which are not obviously involved in any information processing that could be related to a nervous system (seed germination, root architecture, pollen germination: Qiu et al. 2020). The argument that their *GABA* receptors show plants to have neuron-like GABA signaling is even less convincing because the plant version of this receptor is not homologous to that of animals (Jaiteh et al. 2016; Pavlovič et al. 2020), with many structural differences. Thus, there is no guarantee (in the absence of direct evidence) that the anesthetics would even bind to the plant GABA receptor, as they bind to the animal version. Recently, the plant neurobiologists acknowledged this GABA-related threat to their claims (Pavlovič et al. 2020, pp. 180-181), where they also admitted, “. . . it is impossible to identify the protein target of an anesthetic on electrical signals in plants.” De Luccia (2012, p. 1166) admitted the same thing. And without any specific information about targets (the ion channels, etc.), all the claims about anesthesia and plant consciousness are speculation.

In summary, plants lack the structural and functional systems required for anesthetization to reveal anything similar to the consciousness of animals and humans. Anesthetics are drugs whose mechanisms are not as “mysterious” as claimed (Baluška et al. 2016; Yokawa and Baluška 2018). Like many other drugs, especially with the effects in the central nervous system, they have multiple molecular targets and complex systemic effects. It is clear, however, that they disrupt the brain-wide coordinated neuronal activity patterns required for conscious experience and action (Kelz and Mashour 2019). This does not, and cannot, happen in plants where the actions of anesthetics can be explained as mere biochemical and biophysical effects. Similarly, if plants were to show biochemical reactions to antidepressant or antipsychotic drugs, we would not tend to believe that they suffer from depression or schizophrenia.

## Conclusion

**Fig. 2:**
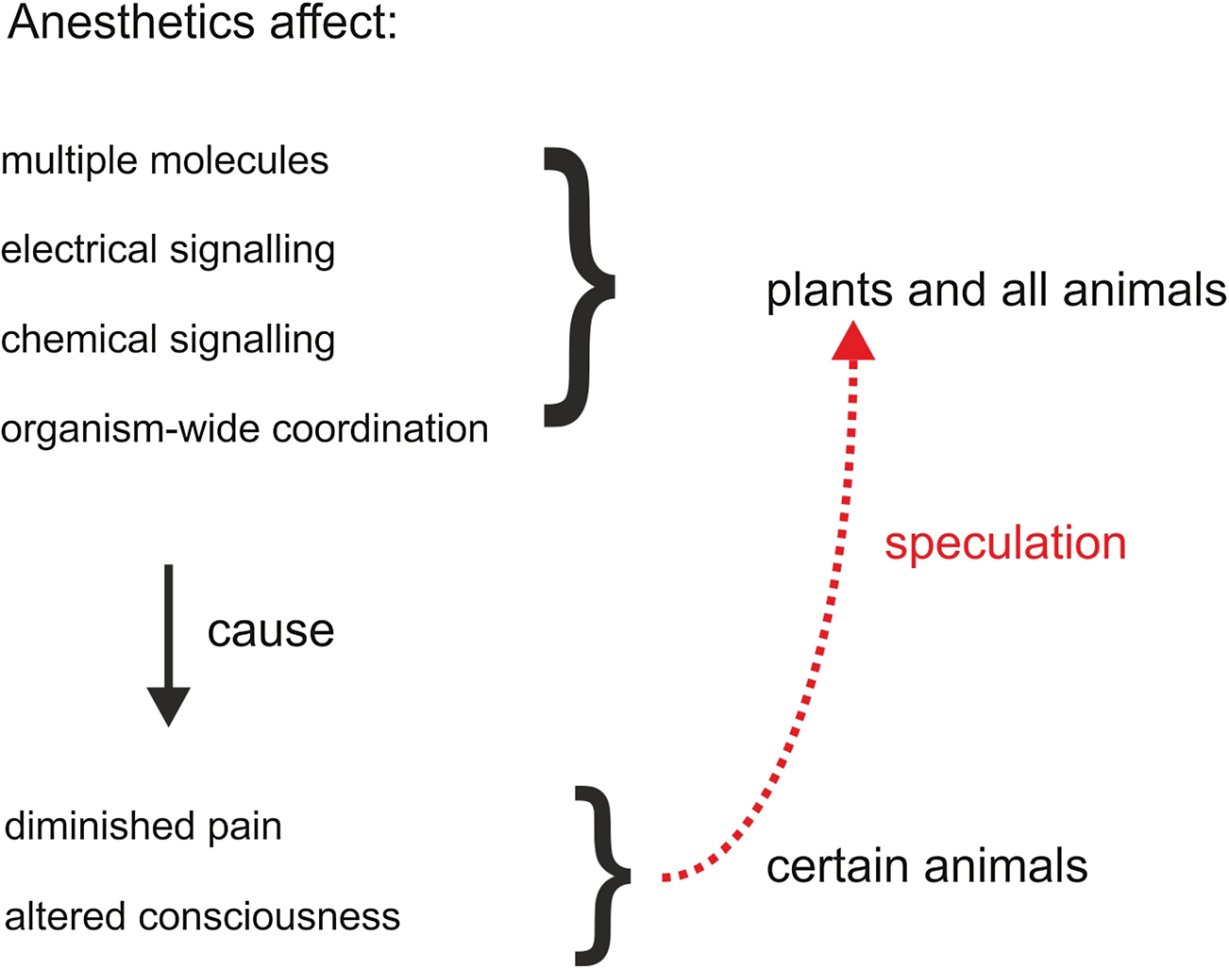
Effects of general anesthetics on plants versus animals. The top half shows the shared effects on plants and on all animals, whereas the bottom half shows that they affect consciousness and pain in certain animals with complex nervous systems. Dotted arrow indicates how plant neurobiologists speculate without evidence that the anesthetics cause the same consciousness-diminishing effects on plants. However, the basic, shared effects can account for plants’ responses without any need to invoke plant consciousness

We conclude that plants do not possess the molecular and structural machinery for pain generation. For anesthetics, there is indeed evidence that these substances affect plants’ non-neural, physiological processes like electrical signaling, growth movements, germination, and multiple biochemical reactions. Taking these effects as evidence for consciousness in plants is, however, an argument without any scientific foundation (Fig. 2). The lack of consciousness also precludes the use of plants as model organisms for studying systemic effects of anesthetics in that context. We therefore cannot agree with Baluška and Reber (2019) who consider the model plant *Arabidopsis thaliana* as an ideal experimental system for studying anesthetics and consciousness. At the very most, studies on plants might aid in unravelling specific molecular or cellular functions of anesthetics, although we are not aware of any prominent example. Studying anesthetics in animals and humans, in contrast, is a flourishing branch of neuroscience and medicine, and all required methods, models, preparations, and conceptual tools are available. From a medical and neuroscience perspective, drugs acting on conscious experience should be foremost studied in organisms possessing consciousness. From a plant-science perspective, experiments on anesthetics in plants do not deliver any information relevant to the question of plant intelligence or consciousness.

## Data Availability

Not applicable.
